# A Comprehensive View of Food Microbiota: Introducing FoodMicrobionet v5

**DOI:** 10.3390/foods13111689

**Published:** 2024-05-28

**Authors:** Eugenio Parente, Annamaria Ricciardi

**Affiliations:** Scuola di Scienze Agrarie, Forestali, Alimentari ed Ambientali, Università degli Studi della Basilicata, 85100 Potenza, Italy; annamaria.ricciardi@unibas.it

**Keywords:** amplicon-targeted metagenomics, microbiota, food, food environments, database

## Abstract

Amplicon-targeted metagenomics is now the standard approach for the study of the composition and dynamics of food microbial communities. Hundreds of papers on this subject have been published in scientific journals and the information is dispersed in a variety of sources, while raw sequences and their metadata are available in public repositories for some, but not all, of the published studies. A limited number of web resources and databases allow scientists to access this wealth of information but their level of annotation on studies and samples varies. Here, we report on the release of FoodMicrobionet v5, a comprehensive database of metataxonomic studies on bacterial and fungal communities of foods. The current version of the database includes 251 published studies (11 focusing on fungal microbiota, 230 on bacterial microbiota, and 10 providing data for both bacterial and fungal microbiota) and 14,035 samples with data on bacteria and 1114 samples with data on fungi. The new structure of the database is compatible with interactive apps and scripts developed for previous versions and allows scientists, R&D personnel in industries and regulators to access a wealth of information on food microbial communities.

## 1. Introduction

Since the first study published in 2009 [1], the use of Next Generation Sequencing to analyze libraries of amplicons of taxonomically relevant regions of the genomes of *Bacteria* and *Archaea* (typically the 16S RNA gene [2,3,4]) and *Fungi* (typically one or more regions including the Internal Transcribed Spacer [5,6,7]) has become the standard tool for the cultivation independent analysis of microbial food communities [8,9,10,11]. The approaches used for sequencing, for the bioinformatic analysis of sequences and for the statistical analysis of the results have been reviewed multiple times [2,12,13,14,15] and the increasing use of third generation platforms is helping in overcoming some of the limitations of short-read sequencing [16,17,18,19,20,21]. Raw sequences are often but not always made available in public repositories, like, among others, the National Center for Biotechnology Information (NCBI) Short Read Archive (SRA, https://www.ncbi.nlm.nih.gov/sra, last accessed on 18 April 2024), the European Nucleotide Archive (ENA, https://www.ebi.ac.uk/ena/browser/home, last accessed on 18 April 2024). In addition, several web-based database and tools [22] provide access to sequences and to processing pipelines. Notable examples are QIITA (https://qiita.ucsd.edu/, last accessed on 18 April 2024) [23], MGnify (https://www.ebi.ac.uk/metagenomics, last accessed on 18 April 2024) [24], IMNGS (https://www.imngs.org, last accessed on 18 April 2024) [25], DBbact (http://dbbact.org/main, last accessed on 18 April 2024) [26], and the Microbe Atlas (https://microbeatlas.org/index.html, last accessed on 18 April 2024) [27]. However, only recently have Minimum Information about MARKer gene Sequence (MIMARKS [28]) standards become compulsory for sample metadata. Among other things, these standards include annotation with a standardized terminology. For food and food environments samples, NCBI SRA incorporates the FoodOn ontology for foods and food processing [29]. Similar controlled ontologies have been developed by the European Food Safety Agency [30]. Unfortunately, older accessions have a variable quality of sample metadata and, even for newer deposits in sequence archives, the quality of metadata is proportional to the good will of researchers. To overcome some of the limitations, we actively develop FoodMicrobionet (FMBN), which up to version 4 only included data on bacterial communities [31,32,33]. Even if the level of sophistication of the database and related software is inferior to other tools, which perform automated searches and analysis on existing repositories or on user-provided projects (like QIITA, MGnify, IMNGS and Microbe Atlas), we feel that the level of annotation of the FoodMicrobionet allows scientists to rapidly access complete information on studies, samples and taxa, integrate them with external resources and rapidly retrieve data for further analysis in a variety of projects. We have demonstrated use cases in a number of recent papers [32,34,35,36,37]. Here, we report on the release of version 5 of FoodMicrobionet, which, for the first time, includes data on fungal microbial communities of food and food environments.

## 2. Materials and Methods

**Figure 1:**
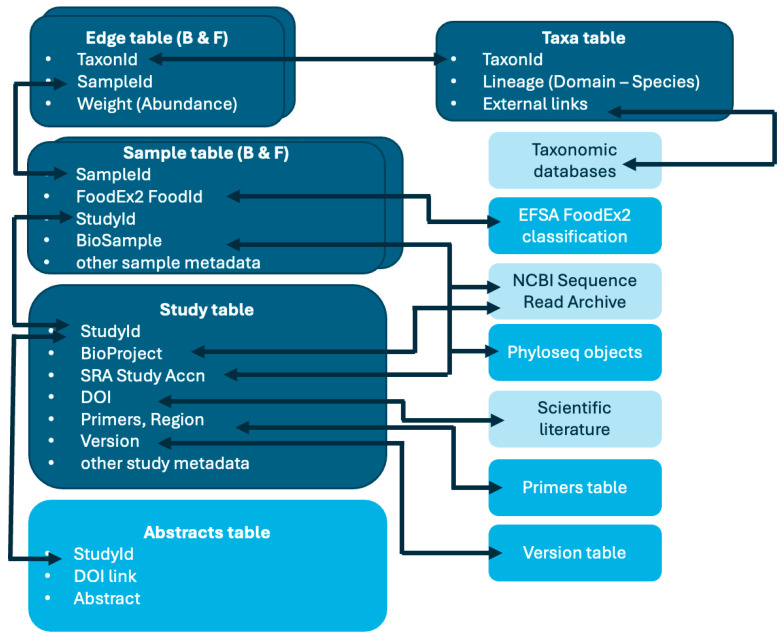
FoodMicrobionet table structure. The main tables are in dark blue; the service tables are in cyan; external resources accessible via links in FoodMicrobionet tables are in light sky blue. Arrows connect primary and foreign key fields in each table.

The structure of FoodMicrobionet v 5.0 has changed little compared to its previous iterations [32,33] and it is schematically shown in Figure 1. 

The main changes include:

1. Addition of data on fungi, with new samples and edges tables;
2. Addition of two further accessory tables, abstracts and version history.

The relational database now includes 4 main tables:

1. Studies: study metadata, including NCBI SRA (https://www.ncbi.nlm.nih.gov/sra, last accessed on 18 April 2024) accession numbers for bioproject and study, references, nucleic acid targets, etc.; each study has a unique id and corresponds to a single NCBI project accession;
2. Samples: sample description, FoodEx2 classification [30], NCBI SRA accession numbers for biosample and run (which are unique for each sample), etc.;
3. Taxa: label and taxonomic lineage for taxa and links to external resources;
4. Edges: relative abundance for each taxon found in a given sample

and a number of service tables:

1. Primers: with sequences of primers used for amplification and target regions;
2. Abstracts: a text table with abstracts for each of the publications from which sequence data were obtained;
3. Version history: change log and references for each FoodMicrobionet version;
4. FoodEx2 classification.

Detailed information on tables and fields is available in Supplementary Material (Supplementary File S1).

To accommodate data on fungal communities of foods and food environments, both the samples and the edges tables were duplicated (i.e., samples_B and samples_F; edges_B and edges_F). This approach was needed for two main reasons:

1. In some cases, data available in NCBI were deposited without fully adhering to SRA specifications (https://www.ncbi.nlm.nih.gov/sra/docs/submitmeta/, accessed on 18 April 2024): i.e., data for the same project were deposited within different bioproject accessions; data for the same biological sample were deposited with different biosample accessions;
2. Metataxonomic taxa abundance data are, in their essence, compositional: no absolute quantification of target for bacteria and fungi is typically available and runs targeting, for example, the V3-V4 region of the 16S rRNA gene for bacteria and Internal Trasncribed Spacer 1 region (ITS1) for fungi are separate. In FoodMicrobionet, for both types of runs, the total number of sequences (which differ in the two runs) and the proportion for each taxon as relative abundance (%) is stored in sample and edges tables, respectively.

The taxa table now includes both bacteria and fungi.

As for previous versions, FoodMicrobionet grows using the addition of raw sequences downloaded from SRA. We regularly scan the literature for new publications and those whose sequence data have been deposited in SRA or ENA are submitted for further screening to evaluate if metadata available in the sequence archives or in the scientific papers enable the obtainment of the information needed to complete the fields in the study and sample table. The raw sequences are then processed with an analysis pipeline adapted from the Bioconductor workflow [38], based on the DADA2 package [39], with several modifications to accommodate for variable length sequences in ITS targeted studies and for sequence data in fastq format with compressed quality scores obtained with the most recent Illumina platforms (including those of the NovaSeq series; https://tinyurl.com/4c3nekt4, last accessed on 23 May 2024). The current version of the pipeline includes options for primer removal with cutadapt [40]. In this version of the database, only studies using the Internal Transcribed Spacer region as a target are used. Taxonomic assignment for fungi is performed using the UNITE [41,42] general release for fungi using the assignTaxonomy() function of the DADA2 package for R [39]. 

After processing, metadata were manually annotated through merging metadata from NCBI SRA with data obtained from the scientific publications. 

A further feature of this version is the addition of a matchId field in sample tables for both bacteria and fungi. This procedure allows rapid identification of matching samples in both tables even when deposit in SRA by the original authors violated the principle that a single biological sample must have a unique accession. 

As in previous versions, all scripts for sequence processing and assembly of the database are publicly available in GitHub (https://github.com/ep142/FoodMicrobionet, last accessed on 23 May 2024).

The database can be easily accessed using an interactive R Shiny app which is publicly available (Parente, Eugenio (2024), “ShinyFMBN, a Shiny app to access FoodMicrobionet”, Mendeley Data, V9, doi: 10.17632/8fwwjpm79y.9), but both the bacterial and fungal versions of the database are compatible, with minor changes, with scripts developed and used in previous proof of concept papers [34,36,43].

## 3. Results and Discussion

### 3.1. FoodMicrobionet, a Global Database

Version 5 of FoodMicrobionet includes data from 251 published studies, 11 of which are on fungal microbiota only, while 230 are on bacterial microbiota. There are 10 studies for which both the data for bacterial and fungal microbiota are available. However, due to inconsistencies in the deposit of sequences in SRA (in several cases the same sample was deposited with two separate biosample accessions and/or data for bacteria and fungi were deposited with different bioproject or study accessions), the same samples were present in two studies, one for bacteria and one for fungi. We did our best to manually match samples in these situations. The addition of datasets on fungi is in progress and, in time, we will add fungal data for all the studies which are already in FoodMicrobionet with bacterial community data and add more studies specialized on fungal microbiota. The combinations of sequencing platforms and targets (Supplementary Tables, Table S1) reflect current and past practices in microbiota analysis. For bacteria, studies performed using the V3-V4 or V4 regions of the 16S RNA gene and with the Illumina platform are the majority. We are currently only adding studies using the ITS target for fungi. Even with its known limitations [20,44], this target is still the most frequently used by the scientific community. On the other hand, FoodMicrobionet is fully transparent in terms of information on targets (which can be selected or excluded in both the studies and sample table) and primers (available in the studies table). This feature is not immediately available in other databases or online tools, like QIITA [23], MGnify [24] or IMNGS [25].

**Figure 2:**
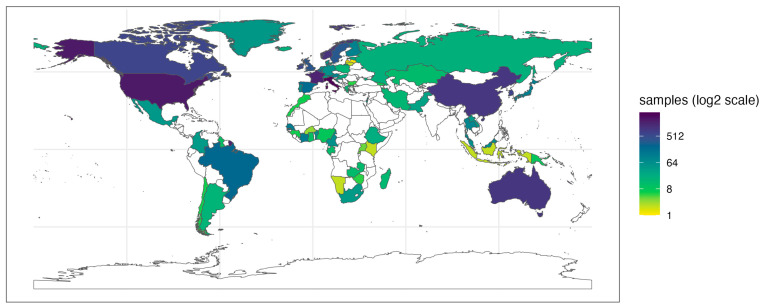
Geographical distribution of samples in FoodMicrobionet 5.0. The continuous color scale for countries represents the number of food and food environmental samples present in FoodMicrobionet for each country.

The latest versions of FoodMicrobionet include information on the geographic location of samples in SRA metadata or in the published papers whenever available. The geographical distribution of the samples is shown in Figure 2.

FoodMicrobionet is a truly global database with samples from 77 countries and 6 continents. The level of detail in the geographical annotation varies, but several samples include geographical coordinate data. This, in principle, would allow interested parties to perform biogeographical studies, comparing the microbiota of a specific product over several locations [45,46]. The level of detail of sample descriptions for both food samples and food environments or food contact surfaces allows the mapping of samples within factories to perform studies of microbial landscapes within a factory or similar factories [47,48].

### 3.2. Diversity of Samples

One of the main strengths of FoodMicrobionet is the level of annotation of sample metadata, which includes a description obtained through incorporating a combination of metadata fields from sample metadata deposited in NCBI SRA, the FoodEx2 [30] code, and the L1, L4 and L6 descriptions from the same classification. Aggregation at other levels of FoodEx2 can be easily performed using the service table FoodEx2. In addition, further fields indicate the type of sample (food or food environment), the stage of the process (raw, intermediate, finished), the use of a lethal treatment, and the occurrence of spoilage and fermentation.

The diversity of samples in FoodMicrobionet is extensive. FoodMicrobionet version 5 includes 14,035 samples with data on bacteria and 1114 samples with data on fungi. For 366 samples, we were able to match samples for bacteria and fungi. There are 2634 environmental samples and 12,125 food samples. Samples in FMBN belong to 19 major food groups (L1 level of FoodEx2 exposure classification, Table 1). Samples in FMBN are further classified using levels L4 and L6 of the FoodEx2 exposure classification, and additional fields (which allow to identify raw products, intermediates or finished products, the level of thermal treatment and the occurrence of spoilage and/or fermentation) allow a finer classification. Samples in FMBN belong to 134 L4 food groups and 239 L6 food groups.

There are 199 foodIds (food types), and, combining further information on samples (nature, heat treatment, spoilage/fermentation), there are 388 combinations. The diversity is particularly extensive for dairy products, including cheeses. 

### 3.3. Accessibility of FoodMicrobionet Data

Researchers with the ability to use the R programming language and environment can make the most of FoodMicrobionet and use it extensively for metastudies to test specific hypotheses or even to obtain quantitative comparisons of their own data with those available in FoodMicrobionet. While we have demonstrated this in previous proof of concept papers [32,34,36,37,49], we will add two common use cases below.

#### 3.3.1. FMBN Made Simple: Accessing Data Using the ShinyFMBN App

**Figure 3:**
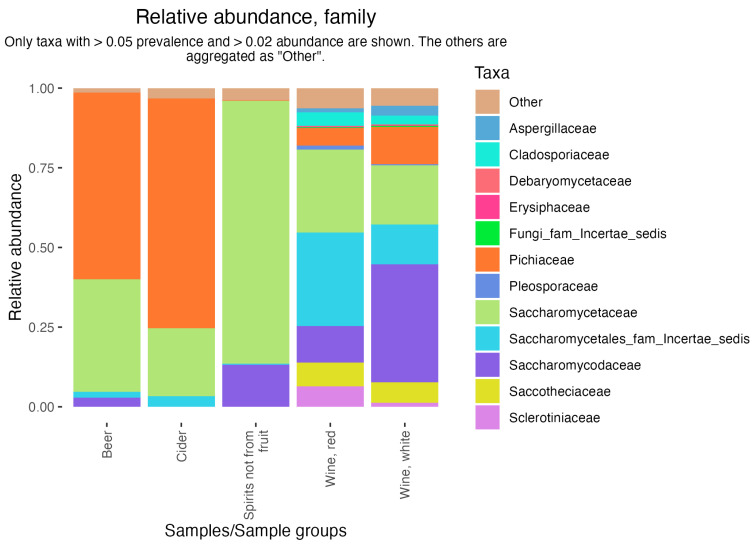
Relative abundance of fungal families in 5 alcoholic beverages. Data extracted from FoodMicrobionet 5.0.

The ShinyFMBN app (https://data.mendeley.com/datasets/8fwwjpm79y/9, last accessed on 23 May 2024) is designed to provide offline access to the database to users who have limited experience with R. The use requires minimal informatic abilities: it is sufficient to install R and launch a script which installs all needed packages and launches the app. By changing a few lines of code in the app, the user can choose to access samples with data on fungi or bacteria. The app can be opened in a browser and allows the exploration of data, the selection of subsets of samples, the export of the subset in several formats (including a phyloseq object [50], a versatile format which is used by many other R packages for the analysis of microbiome data, including the interactive ShinyPhyloseq app [51]). However, ShinyFMBN has also limited capabilities to produce tabular and graphical analysis (prevalence and abundance plots and tables, bar plots, box plots). Figure 3 shows an example: data from six studies on alcoholic beverages [52,53,54,55,56,57] were used to show the distribution of fungal families in beer, wine, cider, pulque, and palm wine. Differences in the relative abundance of yeasts are clearly evident, with beer dominated by *Dekkera* and *Saccharomyces*, cider by *Pichia*, *Saccharomyces*, *Dekkera*, and *Brettanomyces*, palm wine by *Saccharomyces* and *Hanseniaspora*, and wines with a wider diversity of genera.

#### 3.3.2. Using FoodMicrobionet to Explore the Distribution of Microbial Groups in Foods: The Case of Endospore-Forming Bacillota

**Figure 4:**
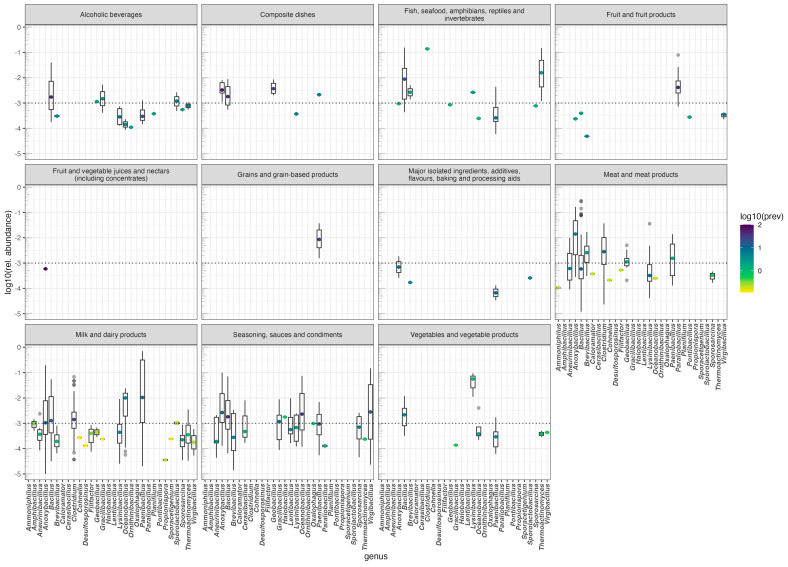
Relative abundance and prevalence of genera of the phylum *Bacillota* which include endospore-forming bacteria in major food groups. Data extracted from FoodMicrobionet 5.0. Median abundance is shown as a dot whose color scale represents the base 10 logarithm of the prevalence (as %) of the genus in a given food group.

We have recently used FoodMicrobionet to explore the distribution of *Lactobacillaceae* in foods [34]. The same approach can be easily used to explore the distribution of other microbial groups which play important roles in the safety and spoilage of foods. Bacteria producing endospores are important in some food fermentations [58] but also contribute to the spoilage of several foods (see, for example [59,60,61,62,63,64]) and some pathogenic species (*Clostridium botulinum*, *C. perfringens*, *Bacillus cereus*) are causative agents of foodborne outbreaks [65,66]. Using the approach used in [34], we extracted data on the relative abundance of endospore-forming Bacillota and used them to build Figure 4. In this figure, the panes (facets) show the distribution of the relative abundance of the main genera in different categories of the L1 level of the FoodEx2 classification. Visual cues on prevalence are also included: The median relative abundance in a given food group is marked by a dot whose color reflects the prevalence. The food groups with the highest diversity of endospore-forming bacteria are milk and dairy products (19 genera), meat and meat products (14) and seasoning and spices (16). While most genera appear in foods with low relative abundance and prevalence, and are possibly contaminants from raw materials or food contact environments and surfaces, others, like *Anoxybacillus*, *Bacillus*, and *Paenibacillus* have a much wider distribution and sometimes their relative abundance exceeds 0.10. Other genera, like *Clostridium* and *Virgibacillus*, are found at relatively high relative abundances and are prevalent only in some foods. The comprehensive annotation of samples in FoodMicrobionet allows the further exploration of these types of data, down to the lowest level of the FoodEx2 classification, providing scientists with a powerful tool to formulate or disprove scientific hypotheses.

## 4. Conclusions

This iteration of FoodMicrobionet provides for the first time both data on bacterial and fungal communities, thus allowing the obtainment of a comprehensive picture of the microbial ecology of foods. With very few changes, the database is retro-compatible with most of the analysis scripts we have developed in the last few years, providing a vast array of statistical and graphic tools for the analysis and representation of the structure of microbial communities. We plan to continue expanding the database by adding data for fungi for existing studies and adding more studies. In addition, we plan to make the ShinyFMBN app available online by using shinyapps.io to eliminate the need for the installation of R.

## Figures and Tables

**Table 1:** Distribution (count, proportion and cumulative proportion) of FoodMicrobionet 5 samples in major categories (L1 level) of the FoodEx2 classification.

L1	n	Prop	Cum.Prop.
Milk and dairy products	5807	0.3931	0.3931
Meat and meat products	3605	0.2440	0.6371
Vegetables and vegetable products	1508	0.1021	0.7391
Fruit and fruit products	1178	0.0797	0.8189
Fish, seafood, amphibians, reptiles and invertebrates	814	0.0551	0.8740
Alcoholic beverages	513	0.0347	0.9087
Major isolated ingredients, additives, flavours, baking and processing aids	339	0.0229	0.9316
Seasoning, sauces and condiments	202	0.0137	0.9453
Grains and grain-based products	155	0.0105	0.9558
Composite dishes	147	0.0099	0.9658
Fruit and vegetable juices and nectars (including concentrates)	82	0.0056	0.9713
Legumes, nuts, oilseeds and spices	81	0.0055	0.9768
Generic food environments	64	0.0043	0.9811
Food products for young population	61	0.0041	0.9852
Eggs and egg products	56	0.0038	0.9890
Animal and vegetable fats and oils and primary derivatives thereof	53	0.0036	0.9926
Sugar and similar, confectionery and water-based sweet desserts	42	0.0028	0.9955
Starchy roots or tubers and products thereof, sugar plants	17	0.0012	0.9983
Coffee, cocoa, tea and infusions	15	0.0010	0.9993

## Data Availability

The database and related scripts are available on GitHub (https://github.com/ep142/FoodMicrobionet, last accessed on 23 May 2024). Additional data (phyloseq objects with ASVs, further metadata) are available on Zenodo (https://zenodo.org/doi/10.5281/zenodo.6954039, last accessed on 23 May 2024). The Shiny app is available on Mendeley data (https://data.mendeley.com/datasets/8fwwjpm79y/9, last accessed on 23 May 2024).

## Supplementary Materials

The following supporting information can be downloaded at: https://www.mdpi.com/article/10.3390/foods13111689/s1, Supplementary File S1: FoodMicrobionet table specifications; Table S1: Distribution of target regions and sequencing platforms for studies in FoodMicrobionet 5.0.
