# Supplementary material for: A Comprehensive View of Food Microbiota: Introducing FoodMicrobionet v5
Source: Foods. 2024 May 28;13(11):1689. doi: 10.3390/foods13111689 (PMC11171936; doi:10.3390/foods13111689)
Supplement: Supplementary file 1 [file foods-13-01689-s001.zip › Supplementary_Tables_S1.pdf]

Communication

A comprehensive view of food microbiota: introducing FoodMicrobionet v5

Eugenio Parente [1,*], and Annamaria Ricciardi [2]

[1] Scuola di Scienze Agrarie, Forestali, Alimentari ed Ambientali, Università degli Studi della Basilicata; eugenio.parente@unibas.it
[2] Scuola di Scienze Agrarie, Forestali, Alimentari ed Ambientali, Università degli Studi della Basilicata; annamaria.ricciardi@unibas.it
* Correspondence: eugenio.parente@unibas.it

Supplementary tables.

Table S1. Distribution of target regions and sequencing platforms for studies in FoodMicrobionet 5.0

		platform		
region	454 GS	Illumina	Ion Torrent	Sum
ITS1	0	6	0	6
ITS1 and V3-V4	0	8	0	8
ITS1 and V4	0	1	0	1
ITS1+ITS2	1	0	0	1
ITS2	0	4	0	4
ITS2 and V3-V4	0	1	0	1
V1-V2	1	2	2	5
V1-V3	33	13	0	46
V2-V3	1	0	0	1
V3	1	3	0	4
V3-V4	1	122	0	123
V4	2	33	3	38
V4-V5	2	5	0	7
V4-V6	0	1	0	1
V5	1	0	0	1
V5-V6	0	1	1	2
V5-V9	1	0	0	1
V6-V8	0	1	0	1
Sum	44	201	6	251
